# Impact of abutment angulation on the accuracy of closed tray versus open tray implant impressions using noble bio-care active implants

**DOI:** 10.6026/973206300211370

**Published:** 2025-06-30

**Authors:** Shaik Riyaz Basha, Shravani Dondla, Praveena Channamsetty, Badugu Satyavathi, Satheesh Simha Reddy, Srivani K., Amisha Agarwal

**Affiliations:** 1 Department of, Malla Reddy Institute of Dental Sciences, Malla Reddy Vishwavidyapeeth, Hyderabad, Telangana, India; 2 Sri Venkata Sai Dental College, Mahbubnagar, Telangana, India; 3 Department of Prosthodontics, Mamata Institute of Dental Sciences, Bachupally, Hyderabad, Telangana, India; 4 Department of Prosthodontics, Malla reddy institute of dental sciences, Malla Reddy Vishwavidyapeeth, Hyderabad, Telangana, India; 5 Department of Prosthodontics, Smilead Dental Care, Hyderabad, Telangana, India; 6 Intern, Kalinga Institute of Dental Sciences, Kalinga Institute of Industrial Technology (KIIT), Deemed to be University, Patia, Bhubaneswar, Odisha, India

**Keywords:** Implant impressions, open Tray technique, closed Tray technique, abutment angulation, procedural score, MM discrepancy, implant accuracy, prosthodontics

## Abstract

The influence of abutment angulation on implant impression accuracy by comparing Closed Tray and Open Tray techniques in a clinical setting is of interest to dentists. Hence, a prospective cross-sectional analysis was conducted on 60 patients receiving implant-supported prostheses, stratified by abutment angulation (0°C, 15°Cand 30-45°C). Impressions were made using polyvinyl siloxane and accuracy was quantified via mean MM discrepancy and procedural performance scores. The Open Tray technique demonstrated superior dimensional fidelity (mean discrepancy = 0.46 mm) versus the Closed Tray (0.72 mm), with significantly higher rates of clinically acceptable impressions and procedural efficacy. Logistic regression identified procedural proficiency as a strong predictor of impression precision. These findings underscore the diagnostic superiority of the Open Tray technique, particularly in angulated scenarios, reinforcing its value in enhancing prosthetic accuracy and clinical predictability.

## Background:

Implant-supported prostheses have revolutionized dental restoration, providing a reliable solution for patients with missing teeth [1, 2]. The accuracy of implant impressions is critical for the successful fabrication and placement of these prostheses. Among various impression techniques, the Closed Tray and Open Tray methods are commonly employed, each with its advantages and limitations [3, 4]. The Closed Tray technique, often simpler and quicker, involves transferring the implant position via an impression copy that remains in place, while the Open Tray method uses a pick-up coping, which is screwed directly into the implant and removed with the Tray, allowing for potentially more precise transfers of implant positions [5]. Abutment angulation plays a significant role in the accuracy of these impressions, as deviations in angle can create challenges in capturing the exact position of the implant in relation to the surrounding anatomy [6]. The Nobel Bio Care Active Implants, known for their advanced design and clinical reliability, were chosen for this study, as they are widely used in contemporary implantology. This study focuses on comparing the Closed Tray and Open Tray impression techniques in terms of their accuracy for implants with varying abutment angulations (0°C, 15°C and 30-45°C). It aims to provide valuable insights into how procedural factors-such as abutment angulation and impression technique-impact the final prosthetic outcome, specifically in terms of positional accuracy. By evaluating the MM discrepancy and procedural scores, this study seeks to determine which impression technique yields the most accurate and reliable results, thus guiding clinical decisions in implant dentistry. Therefore, it is of interest to assess the effect of implant angulation on impression accuracy, which has been underexplored in existing literature.

## Materials and Methodology:

This hospital-based prospective comparative cross-sectional study was conducted over six months in the Department of Prosthodontics and Implantology. The primary objective was to evaluate the impact of abutment angulation on the accuracy of implant impressions using Closed Tray and Open Tray techniques in an in-patient clinical setting, employing Nobel Bio Care Active implants. A total of 60 implant patients aged between 20 and 60 years were recruited based on specific inclusion and exclusion criteria. Eligible patients required implant-supported prostheses with abutment angulations ranging from 0° to 45° and were classified as ASA I or II, indicating good systemic health. Patients with uncontrolled systemic diseases, active periodontal disease, or edentulism and those with implants placed more than six months prior were excluded from the study. Participants were equally divided into two groups: Group A underwent the Closed Tray impression technique, while Group B received the Open Tray technique. Each group further included sub-categories based on abutment angulation (0°C, 15°C and 30-45°C) for comparative analysis. Preoperative evaluation included a comprehensive clinical examination, CBCT imaging to determine implant position and angulation and periodontal health assessment. All implants were placed using standard protocols and abutments were selected based on the planned angulation, confirmed radio graphically. Impressions were taken three months post-implant placement. For the Closed Tray group, plastic impression copings were used and repositioned after removal, whereas the Open Tray group used pick-up type copings secured and removed along with the Tray. Polyvinyl siloxane (light and heavy body) was the material of choice and either custom or perforated Trays were selected according to group assignment. Implant analogs were attached and definitive casts were poured using Type IV dental stone. Accuracy was assessed in two ways. First, MM discrepancy-defined as the positional difference in millimeters between intraoral implant positions and corresponding model analogs was measured using a digital caliper and intraoral scanner by an examiner blinded to the impression technique. MM discrepancy was categorized as good (< 0.5 mm), fair (0.5-1.0 mm), or poor (> 1.0 mm). Second, each impression was scored using a standardized 20-point procedural assessment based on clinical parameters such as angulation, bone density, implant torque and periodontal health. These procedural scores were then classified as good (≥11), fair (7-10), or poor (<7) (Table 1 and 2 - see PDF). Data were analyzed using SPSS version 25.0. An independent t-test was performed to compare the accuracy between the Closed Tray and Open Tray groups. Binary logistic regression was applied to evaluate whether higher procedural scores were predictive of better impression accuracy. The receiver operating characteristic (ROC) curve was plotted to validate the predictive performance of the procedural scoring system. A p-value of less than 0.05 was considered statistically significant (Table 3 - see PDF).

## Results:

A total of 60 patients were evaluated, divided equally into two groups: Group A (Closed Tray) and Group B (Open Tray), each with 30 participants. Implant abutment angulations were further sub-grouped into 0°C, 15°C and 30-45°C for analysis. The results demonstrated that the Open Tray technique had a mean MM discrepancy of 0.46 mm (SD ±0.18), classified as Good accuracy, while the Closed Tray technique showed a higher mean discrepancy of 0.72 mm (SD ±0.21), falling into the Fair category. Statistical analysis using the independent t-test yielded a t-value of -2.11 with a p-value of 0.041, indicating a statistically significant difference between the two techniques (p < 0.05). Clinically, this implies that the Open Tray impression method provides superior positional accuracy, making it a more reliable choice in cases with varying abutment angulations (Table 4 - see PDF). The mean positional discrepancies (in mm) for both techniques were assessed. The Open Tray group demonstrated superior accuracy. The categorical MM discrepancy analysis showed that 60% of impressions in the Open Tray group achieved Good accuracy (<0.5 mm) compared to only 26.7% in the Closed Tray group, while Fair accuracy (0.5-1.0 mm) was observed in 33.3% of Open Tray and 60% of Closed Tray impressions. Notably, Poor accuracy (>1.0 mm) was minimal in both groups but still lower in the Open Tray group (6.7%) than in the Closed Tray group (13.3%). These numerical differences highlight a clinically relevant advantage of the Open Tray technique, indicating it consistently yields more accurate implant impressions, especially in angulated abutment scenarios. The distribution strongly supports the Open Tray method as the more reliable option in achieving prosthetic precision (Table 5 - see PDF).

The procedural score distribution revealed that 56.7% of Open Tray impressions achieved a Good score (≥11), significantly higher than the 30% in the Closed Tray group. Conversely, 56.7% of Closed Tray impressions were classified as Fair (7-10), compared to 36.7% in the Open Tray group. The Poor scores (<7) were minimal in both groups, with 13.3% in Closed Tray and 6.7% in Open Tray. These results suggest that the Open Tray technique not only yields more accurate impressions but also performs better in clinical procedures, demonstrating higher overall quality and fewer instances of suboptimal outcomes. The Open Tray method's superior procedural score reinforces its clinical reliability, especially in cases requiring precise implant positioning (Table 6 - see PDF). Procedural scores were calculated for all impressions based on clinical parameters. Logistic regression analysis demonstrated that the procedural score was a significant predictor of impression accuracy, with an odds ratio of 1.47 (95% CI: 1.03 - 2.11), indicating that for each unit increase in procedural score, the likelihood of achieving a Good MM discrepancy classification increased by 47%. The Area under the ROC Curve (AUC) was 0.70, which suggests moderate predictive accuracy of the procedural score in determining impression quality. With a p-value of 0.032, this result is statistically significant, confirming that higher procedural scores are associated with a greater likelihood of achieving Good impression accuracy. Clinically, this underscores the importance of optimizing procedural factors in achieving high-quality implant impressions, particularly when angulations and other clinical variables are involved (Table 7 - see PDF). Procedural score was a significant predictor of impression accuracy. ROC analysis confirmed that higher procedural scores were associated with greater likelihood of a "Good" MM Discrepancy classification. The study found that the Open Tray technique significantly outperformed the Closed Tray technique in terms of impression accuracy (p = 0.041), demonstrating superior precision. Furthermore, a higher procedural score was strongly associated with better impression outcomes, emphasizing the importance of clinical factors in achieving optimal results. Additionally, implant angulation greater than 30° was linked to poorer procedural scores and MM accuracy, particularly in the Closed Tray group, indicating that increased angulation poses greater challenges for impression accuracy and procedural success. These findings highlight the critical role of technique and procedural quality in ensuring high-accuracy implant impressions, especially in cases involving challenging implant angulations.

## Discussion:

The present study evaluated the accuracy of implant impressions using the closed tray and open tray techniques in patients with varying abutment angulations (0°C, 15°C, 30-45°C). The results revealed that the open tray technique consistently outperformed the closed tray method in terms of both positional accuracy and procedural quality, with statistically significant differences in MM discrepancy (p = 0.041) and procedural scores. These findings align with existing literature, which emphasizes the Open Tray technique's ability to provide more precise implant positioning, particularly in cases where abutment angulation is significant [7, 8]. In contrast, Humphries *et al.* reported that the closed Tray technique demonstrated a stronger correlation with the coordinate measurements on the definitive cast compared to the open Tray technique [9]. The results suggest that the open tray technique's superior accuracy is attributable to its method of capturing the exact position of the implant by securing the impression coping with the Tray. This allows for a more reliable transfer of the implant position, as the coping is retained even during Tray removal, thus minimizing potential errors [10]. Conversely, the closed tray technique, where the coping remains inside the impression during tray removal, often leads to slight positional discrepancies due to the inherent risk of slight movements of the coping during the removal process. This has been a long-standing concern, especially when implants are placed at angulations greater than 30°. The higher mean MM discrepancy and increased incidence of Fair and Poor classifications in the closed tray group further corroborate this, aligning with previous studies that have identified closed tray as less accurate when higher angulation is involved [11]. Furthermore, the procedural score analysis highlighted a critical aspect of impression success: clinical parameters such as implant angulation, bone density and torque significantly affect the impression outcome. Higher procedural scores were associated with better impressions, reinforcing the clinical importance of optimizing these factors [12, 13-14].

The open tray technique, with its better procedural performance, seems to be a more reliable choice in ensuring high-quality impressions when these factors are optimized. This is consistent with literature by Tafti *et al.* (2019), who noted that the Open Tray method excels in clinical situations involving challenging implant placements, particularly with angulated implants [15]. An interesting finding of this study was the impact of implant angulation on procedural success, specifically for angulations greater than 30°. This group experienced more challenges in achieving precise impressions, particularly with the Closed Tray technique, where such angulations likely complicate the stability of the impression coping. This reported in literature that abutment angulation directly affects impression accuracy, with more pronounced discrepancies noted at steeper angles [16]. This is particularly evident in our findings, where Closed Tray impressions for higher angulations exhibited poor procedural scores and a higher frequency of Fair accuracy classifications. Therefore, clinicians should consider these factors when selecting the appropriate impression technique, particularly for implants with significant angulation. In comparison to other studies in the literature, our results are consistent with findings from Jemt *et al.* who concluded that the Open Tray technique provides more accurate results for implants with significant angulation, especially in complex clinical scenarios [17]. However, our study extends these findings by introducing the concept of procedural scoring as a useful predictive tool for clinical decision-making, offering additional insight into the potential for improving impression outcomes by optimizing clinical factors.

## Mode of procedure complications:

Despite the clear advantages of the Open Tray technique, there are inherent complications associated with its use. One common issue is patient discomfort during the impression-taking process, especially when implants are located in difficult-to-reach areas of the mouth. The process of screwing the coping into the implant can be more invasive, which may result in increased patient discomfort compared to the Closed Tray method. Additionally, the Open Tray technique requires more precise Tray placement and handling, increasing the risk of procedural errors if not executed carefully. However, the improved accuracy justifies the extra effort, especially in challenging cases [18, 19-20]. Closed Tray impressions, on the other hand, are quicker and less invasive, making them a preferable option in cases where comfort and speed are priorities. However, as our results show, Closed Tray impressions are more susceptible to positional errors in angulated implant placements, leading to poorer accuracy and prosthetic fit. For Nobel Active implants, Hazboun *et al.* investigated the impact of closed and open tray impression procedures *in vitro* and found that implant angulation and impression technique (open vs. closed tray) had no discernible effects on *in vitro* impressions [21]. According to Conrad *et al.* there was no discernible difference in the average angle errors between the closed and open tray impression processes [22]. The accuracy of implant impressions was not affected by the sub-gingival depth of implant placement, according to Lee *et al.*'s evaluation of the subject [23]. Moreover, it is essential to consider that while the Closed Tray method offers ease of use, its lower reliability for angulated implants could result in the need for repeated impressions or adjustments during the restorative phase, ultimately increasing treatment time and costs [20]. Our results further support these observations by demonstrating that higher procedural scores significantly correlate with improved MM accuracy, underscoring the value of clinical expertise and optimal procedural execution in achieving the best outcomes. In conclusion, this study confirms that the Open Tray technique is superior in terms of positional accuracy and procedural performance, particularly for implants with significant angulation. However, the Closed Tray technique may still have a place in clinical practice, especially when ease of use and patient comfort are prioritized. It is essential for clinicians to consider implant angulation, procedural factors and patient needs when selecting the most appropriate impression technique. Future research should explore more advanced methods for improving the Closed Tray technique and evaluating the long-term outcomes of both methods in various clinical settings.

## Limitation:

This study was conducted with a relatively small sample size, which may limit the generalizability of the results to a larger population.

## Future perspective:

Future studies could explore the impact of different impression materials and advanced technologies on the accuracy and reliability of both impression techniques in a larger cohort.

## Conclusion:

The Open Tray technique significantly out-performed the Closed Tray method in achieving superior impression accuracy, especially in challenging implant angulations. Higher procedural scores were strongly predictive of better outcomes, emphasizing the critical role of technique and clinical precision in ensuring successful implant prosthetics.

## References

[1] Gowd M.S (2017). J Int SocPrev Community Dent..

[2] Duong H.Y (2022). Periodontol 2000..

[3] Fathi A (2023). Clin Exp Dent Res..

[4] Flügge T (2018). Clin Oral Implants Res..

[5] Agarwal S (2020). J Long Term Eff Med Implants..

[6] Kanneganti K.C (2018). J Indian Prosthodont Soc..

[7] Tafti A.F (2019). Dent Res J (Isfahan)..

[8] Dohiem M.M (2022). BMC Oral Health..

[9] Humphries R.M (1990). Int J Oral Maxillofac Implants..

[10] Yang X (2022). J Prosthet Dent..

[11] Hsu C.C (1993). J Prosthet Dent..

[12] Sreerama R (2021). J Pharm Bioallied Sci..

[13] Jo S.H (2010). J Adv Prosthodont..

[14] Barone A (2016). Clin Implant Dent Relat Res..

[15] Tafti A.F (2019). Dent Res J (Isfahan)..

[16] Sorrentino R (2010). Clin Implant Dent Relat Res..

[17] Tandon A (2018). Journal of Clinical and Diagnostic Research..

[18] Carr A.B (1991). Int J Oral Maxillofac Implants..

[19] Hatim N.A (2007). Al. Rafidain Dent J..

[20] Balouch F (2013). J Dent (Shiraz)..

[21] Hazboun G.B.A (2015). J Prosthet Dent..

[22] Conrad HJ (2007). J Prosthet Dent..

[23] Lee H (2008). J Prosthet Dent..

